# Simultaneous Quantification of Main Saponins in *Panax vietnamensis* by HPLC-PDA/ELSD Using the Quantitative Analysis of Multi-Components by Single-Marker Method

**DOI:** 10.3390/metabo15070419

**Published:** 2025-06-20

**Authors:** Thi-My-Duyen Ngo, Thi Kim Ngan Tran, Thi Minh Thu Le, Mong Kha Tran, Huu Son Nguyen, Huy Truong Nguyen, Kim Long Vu-Huynh

**Affiliations:** 1 Faculty of Pharmacy, Ton Duc Thang University, Ho Chi Minh City 700000, Vietnam; ngothimyduyen@tdtu.edu.vn (T.-M.-D.N.); kimngantran0503@gmail.com (T.K.N.T.); tranmongkha@tdtu.edu.vn (M.K.T.); nguyenhuuson@tdtu.edu.vn (H.S.N.); 2 Faculty of Pharmacy, University of Medicine and Pharmacy at Ho Chi Minh City, Ho Chi Minh City 700000, Vietnam; ltmthu.ncs.kntdc24@ump.edu.vn; 3 Research Group in Pharmaceutical and Biomedical Sciences, Faculty of Pharmacy, Ton Duc Thang University, Ho Chi Minh City 700000, Vietnam; nguyentruonghuy@tdtu.edu.vn

**Keywords:** HPLC-PDA, HPLC-ELSD, *Panax vietnamensis*, QAMS, relative conversion factors, saponin, majonoside R2, ocotillol

## Abstract

**Background**: The Quantitative Analysis of Multi-components by Single-marker (QAMS) method has been developed as an alternative to the External Standards Method (ESM) for the quality control of medicinal herbs. **Objectives**: In this study, QAMS was developed to determine saponins in the raw materials of *Panax vietnamensis* using HPLC-PDA/ELSD. **Methods**: The method was developed and validated. The relative conversion factors F_x_ were calculated based on the linear regression for HPLC-PDA and the logarithm equation for HPLC-ELSD. The Standard Method Difference (SMD) was determined to indicate the difference in the results of QAMS and EMS. **Results**: Relative conversion factors (F_x_) were determined for each detector to quantify five saponins (ginsenoside Rb_1_, Rd, Rg_1_, majnoside R2, and vina-ginsenoside R2) in VG root. The F_x_ values were calculated based on the ratio of the slopes of the regression equations of a single standard and an external standard. For HPLC-PDA, G-Rb1 was used as a single standard with the F_x_ values of 1.00 (G-Rb_1_), 1.08 (G-Rd), 1.32 (G-Rg_1_), and 0.04 (M-R2). For HPLC-ELSD, G-Rb_1_ was used for determining the content of G-Rg_1_ and G-Rb_1_ with the F_x_ values of 1.00 (G-Rb_1_) and 0.95 (G-Rg_1_), while M-R2 was used for quantitating M-R2 and V-R2 with F_x_ of 1.00 (M-R2) and 1.05 (V-R2). An SMD value less than 5.00% confirms the close alignment of the QAMS method with ESM. **Conclusions**: The QAMS method proved to be a feasible and promising method for the quality control of *P. vietnamensis*.

## 1. Introduction

**Figure 1:**
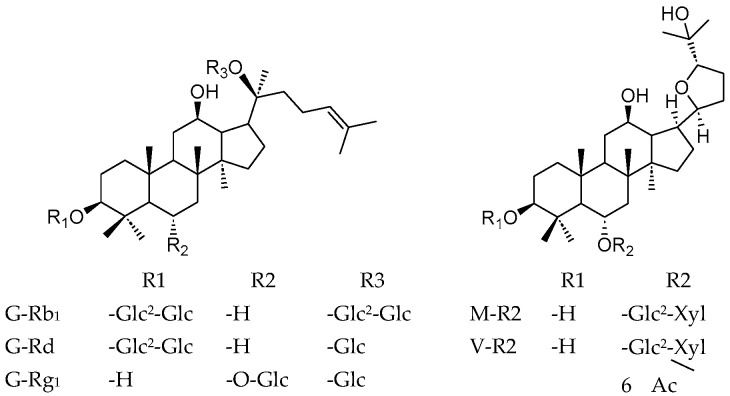
Structure of major saponins in Vietnamese Ginseng.

*Panax vietnamensis* Ha *et* Grushv. (Araliaceae), commonly known as Vietnamese Ginseng (VG), is considered a precious medicinal herb and a national treasure of Vietnam. Numerous investigations indicate that the dammarane saponin constituents of VG are similar to those of other *Panax* species, such as Korean Ginseng (*P. ginseng*), Sanchi (*P. notoginseng*), and American Ginseng (*P. quinquefolius*). These saponins include protopanaxadiol (PPD) (ginsenoside Rb_1_, Rb_2_, Rd, etc.) and protopanaxatriol (PPT) (G-Re, -Rg_1_, etc.). Notably, VG contains a high content of ocotillol-type saponins (OTs) such as majonoside R2 (M-R2) and vina-ginsenoside R2 (V-R2), etc. (Figure 1), which are unique among *Panax* species and represent the major saponin group in *P. vietnamensis*. Among these characteristic compounds, M-R2 is the predominant saponin with a content of over 5.0% [1]. Due to its distinct chemical profile and therapeutic benefits, VG has garnered considerable attention from consumers. Consequently, ensuring the quality of raw materials and preparations derived from VG has become a significant concern, particularly with regard to its saponin content.

Modern analytical techniques, such as High-Performance Liquid Chromatography (HPLC) and Gas Chromatography–Mass Spectrometry (GC-MS), are widely employed to control the quality of herbal materials. These methods generally require reference standards for identification and quantification [2]. However, reference standards are often expensive, not readily available locally, and must primarily be imported. As a result, the traditional quantification method based on the External Standards Method (ESM) faces challenges in cost and time efficiency. The Quantitative Analysis of Multi-Components by Single-marker (QAMS) method has emerged as a promising alternative to address these issues. This method was first developed in 2006 to evaluate the quality of Chinese herbal medicines [3] and has since been adopted in numerous pharmacopeias worldwide for the effective and accurate quality control of herbal materials. For example, in USP-NF 2024, the monograph of Chinese *Salvia* prescribes the use of a tanshinone IIA standard for the simultaneous quantification of cryptotanshinone, tanshinone I, and tanshinone IIA; *Radix et Rhizome Rhodiola crenulata* uses salidroside for the quantification of salidroside and tyrosol [4]. In EP 11.2, *Ginkgo* dry extract, a refined and quantified monograph, uses an external standard, benzyl alcohol, which is not included in the preparation, for the simultaneous quantification of four terpenoids (bilobalide, ginkgolide A, ginkgolide B, ginkgolide C) [5]. In addition, many studies have applied QAMS for quantitative analysis in herbal materials, such as using ganoderic acid A as a single standard for ten components, Lingzhi [6], and using echinacoside as a standard for the quantification of seven phenylethanoid glycosides in *Cistanches herba* [7]. Many studies have been carried out using QAMS for the quantitative determination of saponins in *Panax* species. Zhu et al. (2008) used G-Rb_1_ for the simultaneous determination of nine ginsenosides in *P. ginseng* and four ginsenosides in *P. notoginseng.* The relative conversion factors (F_x_) were determined for the eight other ginsenosides with good reproducibility in different HPLC instruments and columns [8]. Wang et al. (2015) investigated the influence of different factors on the accuracy of the QAMS method in the quantitative analysis of saponins in *P. notoginseng*. The research figured out that the concentration of the analytes was the key parameter influencing the precision of the QAMS method. As a result, a linear regression-based method for calculating the F_x_ was established. The proposed method proved to significantly decrease the standard method differences (SMD) [9]. It is notable that the saponins in these two ginsengs are in the PPT and PPD skeletons with one double bond in the structure. Therefore, a UV or Photodiode Array (PDA) detector is usually used due to its popularity and affordable price. However, the other ocotillol-type saponins, such as pseudoginsenoside F11 or M-R2, without double bonds in the structure, exhibit a challenge in detection with a UV detector, even at very low wavelengths. In this case, a universal detector such as Evaporating Light Scattering Detector (ELSD), Charged Aerosol Detector (CAD), or Mass Spectrometry (MS) showed its strength in detecting the non-UV absorption compounds [10]. The CAD is also a cost-effective alternative to the expensive MS detector for detecting compounds lacking double bonds in their structures. Yao et al. (2021) established an HPLC-CAD with the QAMS method using astragaloside I to determine four non-chromophore saponins (astragaloside I, astragaloside II, astragaloside IV, calycosin-7-*O*-β-*D*-glucoside) in *Astragalus membranaceus* [11].

Given the advantages of QAMS in terms of convenience, cost-effectiveness, and alignment with international harmonization in testing, we propose developing a quantitative method for saponins in *Panax vietnamensis* using HPLC-PDA/ELSD based on the QAMS method. In addition to the PDA detector, we also used an ELSD to optimize the detection of ocotillol-type saponins (OTs), which are difficult to detect with a PDA due to the lack of chromophore in the structures, despite being the main components. The newly developed QAMS method was validated and assessed by comparing its results with those obtained using the ESM.

## 2. Materials and Methods

### 2.1. Materials and Chemicals

The reference substances were primary standard compounds (P), including G-Rb_1_ (99.17%), G-Rd (94.48%), G-Rg_1_ (96.43%), M-R2 (98.86%), and V-R2 (97.63%), supplied by the University of Medicine and Pharmacy at Ho Chi Minh City, Vietnam.

The roots of VG were collected from Tra Linh Mountain, Nam Tra My District, Quang Nam Province. The fresh roots were thoroughly cleaned, sliced, and dried at 60 °C until dry. The dried roots were ground into fine powder with a particle size of less than 1 mm.

### 2.2. Preparation of Sample Solutions

An accurate weight of 100 mg of VG powder was placed into a 15 mL tube and extracted three times, each for 20 min by ultrasound, with 5 mL of 80% methanol. The extract was centrifuged at 4000 rpm for 5 min, and the supernatant was combined and evaporated to dryness. The residue was dissolved in 6 mL of distilled water and loaded into an SPE-C18 column preconditioned with 6 mL of 100% methanol, followed by 12 mL of distilled water. The polar impurities were removed using 6 mL of water, and the saponins were eluted with 4 mL of methanol into a 5 mL volumetric flask and then filled up to the volume with the eluting solvent. The test solution was strained through a 0.45 µm pore size filter and was used as the sample solution [12].

### 2.3. Preparation of Ginsenoside Standard Solutions

Dissolve the standard compounds G-Rb_1_, G-Rd, G-Rg_1_, M-R2, and V-R2 in 80% methanol to obtain the C1 standard solution, with concentrations of 0.36 mg/mL, 0.17 mg/mL, 1.39 mg/mL, 2.30 mg/mL, and 0.81 mg/mL, respectively. Serially dilute the C1 solution to obtain standard solutions with concentrations of C2 to C6, using a dilution factor of 0.75.

### 2.4. HPLC-PDA/ELSD Parameters

A Shimadzu Prominence-I LC-2030C 3D Plus system, coupled with a PDA detector and Sedex 85 LT-ELSD and an Agilent 1260 Infinity II HPLC-DAD system, was used for HPLC-PDA/ELSD analysis. The saponins were separated by a Phenomenex Luna C18 column (150 mm × 4.6 mm, 5 μm). The mobile phase consisted of acetonitrile (A) and water (B) with the following gradient program: 0–25 min, 23% A; 25–35 min, 23–32% A; 35–50 min, 32% A; 50–68 min, 32–35% A; 68–75 min, 35–95% A; 75–76 min, 95–23% A; 76–86 min, 23% A. The injection volume was 25 μL, with a flow rate set at 0.8 mL/min. The column temperature was maintained at 30 °C. The PDA detector was set at 196 nm, while the Sedex 85 LT-ELSD detector was set up with a temperature of 40 °C and a gas pressure of 340 kPa, and nitrogen was used as the nebulizing gas.

### 2.5. HPLC-PDA/ELSD QAMS Method Establishment

The equations (Eq.) for the QAMS methods are shown in Table 1 [13].For HPLC-PDA, the ESM involved constructing a linear regression equation (Equation (1)). In contrast, the QAMS method determined the relative conversion factor (F_x_) using Equations (2) and (3). For HPLC-ELSD, logarithmic Equation (7) was applied to calculate the analyte concentration based on the peak area (A_x_). The average F_x_ values were then calculated using two approaches: the ratio of the peak area’s logarithm to the concentration’s logarithm (Equation (8)) and the ratio of slopes (Equation (9)). The saponin concentration (C_x_) was calculated using Equations (4)–(6), corresponding to Equations (1)–(3). Similarly, for Equations (7)–(9), C_x _was determined using Equations (10)–(12), respectively. From the concentration C_x_, the content of saponin in the dried material can be calculated using the equation: H(%) = (V × C)/m × 100, where C is the concentration of saponin in the sample solution (mg/mL), V is the volume of the sample solution (5 mL), and m is the weight of the dry sample (mg).

The ESM was utilized to assess the accuracy of the QAMS method, with the standard method difference (SMD) determined using Equation (13):

(13) $$\mathrm{SMD}\;(\%)=\left|H_{\mathrm{ESM}}-H_{\mathrm{QAMS}}\right|{/H}_{\mathrm{ESM}}\times 100,$$

where H_ESM_ and H_QAMS_ represent the saponin content in an analyte assayed by the ESM and QAMS method, respectively. The saponin content variations between the QAMS method and ESM within 5.00% indicate no significant difference in the results obtained by the two methods [14,15]. The HPLC method was validated in accordance with ICH [16] and AOAC guidelines [17], covering specificity, linearity and range, limits of detection and quantification, repeatability, and accuracy.

## 3. Results

### 3.1. Relative Conversion Factor Calculation

When utilizing a single marker to quantify multiple components in a sample, it is mandatory that the reference standards are readily available, stable, and effectively separated from other compounds under the specified chromatographic conditions. This study selected G-Rb_1_ as a single marker for the indirect quantification of five target saponins (G-Rb_1_, G-Rd, G-Rg_1_, M-R2, and V-R2) due to its high content, ease of isolation, stability, and clear chromatographic signals on both the PDA detector and ELSD. Given that the distinction between PPD- and PPT-type saponins lies solely in the number and position of hydroxyl (-OH) groups, G-Rb_1_ was chosen to establish the relative conversion factor for both types. For OT-type saponins, this study utilized a combination of G-Rb_1_ and M-R2 for quantification to compare the method’s accuracy. F_x_ was established based on the mean calibration curve, calculated from three linear calibration curves of the reference standards obtained over three separate experimental days. The results are presented in Table 2.

### 3.2. Method Validation of HPLC-PDA/ELSD Methods

#### 3.2.1. Specificity

**Figure 2:**
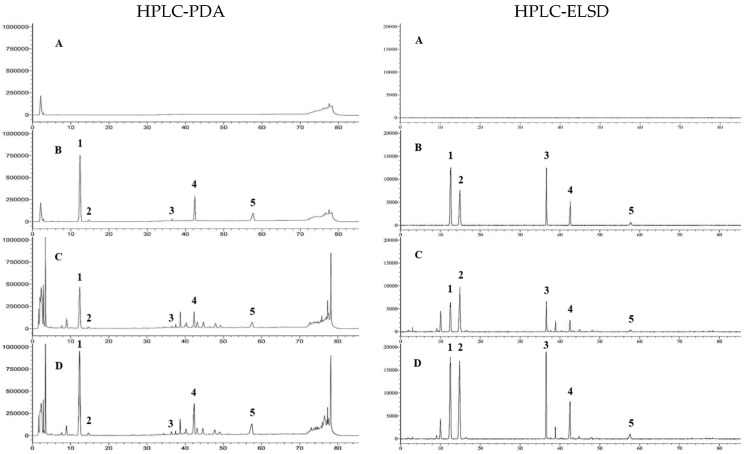
The specificity of HPLC-PDA and HPLC-ELSD methods. A: blank; B: standard; C: sample; D: spiked sample. 1: G-Rg_1_; 2: M-R2; 3: V-R2; 4: G-Rb_1_; 5: G-Rd.

The blank, standard, sample, and sample solutions with added reference standards were all injected under the same HPLC conditions. The results of specificity are displayed in Figure 2.

The retention times of the saponins in the standard and sample solutions were identical (see Supplementary Table S1). In the chromatogram of the blank solution, no peaks were observed at the retention times corresponding to those of the standard solution. In the chromatogram of the sample spiked with the standard, the peak areas of the major saponins under investigation increased compared to those in the unspiked sample. Thus, the quantitative analysis method for saponins in *Panax vietnamensis* using HPLC-PDA and HPLC-ELSD demonstrated specificity.

#### 3.2.2. Linearity, Linear Range, Limit of Detection, and Limit of Quantification

The linearity, correlation coefficients (*R*), linear range, limit of detection (LOD), and limit of quantification (LOQ) for the five saponins are summarized in Table 3. The calibration curves showed good linearity within the selected concentration ranges, with correlation coefficients (*R* > 0.9990), except for G-Rd in the HPLC-ELSD method. Moreover, the concentration of G-Rd in the sample solution did not meet the LOQ, leading to calculation errors. Therefore, the HPLC-ELSD method was applied to quantify only four saponins: G-Rb_1_, G-Rg_1_, M-R2, and V-R2. The V-R2 compound exhibited low sensitivity when analyzed with the PDA detector, as illustrated in Figure 1 with S/N =2.6. The minimal response of this compound is due to the absence of double bonds in its structure, which leads to the poor absorption of UV light even at a very low wavelength (196 nm). Therefore, the PDA detector was used to quantify four saponins: G-Rb_1_, G-Rd, G-Rg_1_, and M-R2.

#### 3.2.3. Precision

Repeatability (intra-day) was assessed by preparing six sample solutions using the same procedure and analyzing them under identical conditions. The saponin contents and their relative standard deviation (RSD) values were calculated. Intermediate precision (inter-day): There are many ways to establish a model to evaluate the intermediate precision (different days, environmental conditions, instruments, etc.). In this study, we conducted inter-day precision by analyzing six sample solutions each time on two different days using a Shimadzu HPLC system. Reproducibility (inter-laboratory): The experiments evaluated reproducibility in the same way as intermediate precision but at two different laboratories using two different HPLC-PDA systems (Shimadzu and Agilent). The saponin contents and their RSD values were calculated. The saponin content was determined using two methods: the ESM and the QAMS method. The RSD values for precision are listed in Table 4.

In the repeatability tests for HPLC-PDA and HPLC-ELSD, all RSD values ranged from 0.26% to 3.05%, demonstrating the reproducibility of the three methods. In the inter-day test for HPLC-ELSD, the RSD values ranged from 1.90% to 2.83% (Table 5). No significant differences were observed in the inter-day results for the saponin content between measurements. In the reproducibility test for HPLC-PDA, the quantitative results for G-Rb_1_, G-Rg_1_, and M-R2 showed no significant differences between the two systems, with RSD values ranging from 1.89% to 3.95%. However, the RSD value for G-Rd was higher than those of the other compounds, approximately 6.47–6.81%. This could be attributed to the relatively low G-Rd content in the sample (approximately 0.9%) and its minimal absorbance on the chromatogram. These factors likely contributed to significant variability in the quantification results when comparing measurements conducted using two different HPLC systems.

#### 3.2.4. Accuracy

Accuracy was assessed using the spiked standard method. Three concentration levels of the five standards (approximately corresponding to 80%, 100%, and 120% of each compound) were added to the sample. For each concentration level, three test samples were prepared and analyzed. The mean recoveries of the five components using HPLC-PDA and HPLC-ELSD were calculated and are displayed in Table 6.

The results indicate that the mean recoveries calculated using ESM Equation (4) are comparable to those obtained with QAMS Equation (6) using HPLC-PDA (79.37–108.45%). Similarly, recoveries calculated using ESM Equation (10) are comparable to those from QAMS Equation (12) using HPLC-ELSD (87.69–111.62%). The RSD values for recovery among the three samples with the same added standard concentration ranged from 0.08% to 4.42%. 

Furthermore, compared to the ESM, the indirect quantification methods using QAMS Equation (5) or Equation (11) exhibited significantly greater variability in recovery rates, ranging from 73.95% to 108.92% for HPLC-PDA and from 74.13% to 191.90% for HPLC-ELSD. This indicates that the deviation in the quantification results using these two methods is larger compared to the results obtained with QAMS Equations (6) and (12).

In summary, the QAMS method using F_x,_ based on the ratio of slopes, produced results more consistent with the ESM than those using F_x_ based on the ratio of A/C or log(A)/log(C).

3.2.5. Comparison of QAMS and EMS methods

In the QAMS method, Fx was calculated using the ratio of slopes, yielding validation results for accuracy and precision closer to those of the ESM than those obtained using the A/C or log(A)/log(C) ratios. However, the quantitative results under different conditions were compared to investigate the possibilities further.

Quantitative analysis with the relative conversion factor (F_x_ intra-day): Table 7 and Table 8 compare the quantification results of saponins using the ESM and the two QAMS methods, with F_x_ values derived from the regression equations established on a single day.

As presented in Table 7, the differences among the three methods (the ESM and two QAMS methods) were insignificant for the PDA detector. However, for the ELSD, the difference between the ESM and QAMS (with F_x_ = log(A)/log(C)) was substantial. For the QAMS method using F_x_ calculated based on the ratio of slopes, the indirect quantification of OT-type saponin using G-Rb_1_ on this detector showed a significant deviation, with the %SMD of M-R2 being 27.65 ± 0.48. In contrast, when M-R2 was used to quantify OT-type saponin, the results were consistent with those obtained using the ESM (Table 8). Therefore, for the indirect quantification of saponin by HPLC-ELSD using F_x_ intra-day, it is recommended to use two markers: G-Rb_1_ for quantifying G-Rb_1_ and G-Rg_1_, and M-R2 for quantifying M-R2 and V-R2.

Quantitative analysis with the average relative conversion factor (F_x_ inter-day): On the HPLC-PDA/ELSD Shimadzu system, F_x_ was calculated based on the standard curve averaged over three different days. The mean content of the five saponins and the SMD values were compared between the ESM and the two QAMS methods, as listed in Table 9 and Table 10.

For the PDA detector, the SMD values did not exceed 2.00% (Table 9). However, when using the ELSD, there was a relative difference in the calculated saponin content when using F_x_ (log(A)/log(C)) compared to the ESM. In contrast, using F_x_ (slope) yielded results that closely matched those obtained with the ESM with the SMD less than 0.05% (Table 10). Therefore, with the F_x_ obtained by the average of F_x_ calculated with Equation (9) on three different days, G-Rb_1_ could be used as the single reference standard for the simultaneous determination of G-Rb_1_, G-Rg_1_, M-R2, and V-R2.

Comparison of quantification results using F_x_ intra-day and F_x_ inter-day values: The above results demonstrate that the QAMS method using F_x_, calculated based on the ratio of slopes, provided results that closely matched those obtained by the ESM. However, the SMD values between the two QAMS (F_x_-Slope) approaches, one using F_x_ calculated from a single day and the other using F_x_ averaged over three days, had not been analyzed. Table 11 presents the SMD values for these two methods, corresponding to the PDA detector and the ELSD.

The quantification results indicate that there is no significant difference in the determination of saponins in the VG samples using intra-day and inter-day F_x_-Slope values (SMD ≤ 5.00%). The data prove the possibility of the use of G-Rb_1_ as the single reference standard for the simultaneous determination of G-Rb_1_, G-Rd, G-Rg_1_, and M-R2 by HPLC-UV and G-Rb_1_ and G-Rg1 by HPLC-ELSD. M-R2 could be used as the second standard to quantify M-R2 and V-R2.

## 4. Discussion

VG (*P. vietnamensis*) is an extremely highly valued medicinal herb on the market. The appearance of VG underground consists of a rhizome and radix, which is quite similar to Sanchi (*P. notoginseng)* or Japanese ginseng (*P. japonicus*). The chemical composition of VG shares the common PPT- and PPD-type ginsenoside with other *Panax* species, such as G-Rb_1_, Rg_1_… However, VG possesses a high content of ocotillol-type saponins such as M-R2 and V-R2 that could distinguish VG from Korean Ginseng or Sanchi. Therefore, the quality control of VG using a comprehensive chemical profile is an urgent matter to ensure the interest of the consumer as well as the reputation of the herb. Hence, the simultaneous quantification of saponins in the three skeletons of PPT (G-Rg_1_), PPD (G-Rb_1_), and OT (M-R2, V-R2) is a strict requirement. However, the traditional ESM is expensive and depends on many reference standards that are hard to acquire, especially the ocotillol-type saponin characterized for VG. Therefore, the development of the QAMS method is an efficient alternative to the common quantitative methods. In this study, a QAMS method employing HPLC coupled with a PDA and ELSD was developed to quantify five major saponins in VG, including G-Rb_1_, G-Rd, G-Rg_1_, M-R2, and V-R2, using G-Rb_1_ and M-R2 as reference standards. The developed method was validated to meet the requirements of international guidelines (ICH) with high consistency with the ESM (SMD below 5%). The developed QASM methods reduce the reliance on multiple reference standards, which could lower the cost of the quality control of VG raw materials and products. This could also enhance the accessibility of the analytical labs, particularly due to the limitation of OT-type saponin standard resources. The high consistency with the ESM ensures that the QAMS method could be a viable candidate for inclusion in future pharmacopeias for effective quality control of VG as well as derived products.

There are many issues concerning the use of equations in the QAMS method. According to research on using the QAMS method for determining triterpene acids in *Ganoderma lucidum*, some issues might become apparent when the A/C value is used (Equation (2)) [6]. Smaller peak areas resulted in a larger variance of A/C, which, in turn, influenced the average value of those ratios, namely the F_x_ value. Because each concentration point has different levels of deviation, when the numerator and denominator correspond to different concentration points, the F_x_ value obtained differs. This issue was supported by the report of Wang et al. (2015) concerning the key role of concentration on the accuracy of the QAMS method [9]. Therefore, F_x_ obtained from the ratio of the slopes from the regression equation gives a reliable QAMS method by avoiding the impact of concentration variance.

Similarly to the previously mentioned study, G-Rb_1_ is also used as the marker for the quantitative determination of PPT- and PPD-type ginsenosides. However, the levels of M-R2 and V-R2 are significantly higher in comparison with G-Rb1. In addition, the detection principle of ELSD totally depends on the number of eluted particles, which is tightly correlated to the compound concentration. Therefore, in the HPLC-ELSD method, the use of G-Rb_1_ as the marker for the quantity of these two OT-type saponins is not appropriate (SMD > 18%). Therefore, the additional use of M-R2 as the second marker, besides G-Rb_1_, makes the method accurate and reliable. Moreover, M-R2 is also available as the reference standard on the market, which facilitates the application of HPLC-ELSD using QAMS for the quality control of VG raw materials and products. Therefore, the developed method could be utilized for the determination of other minor OT-type saponins in VG, such as vina-ginsenoside R11, pseudoginsenoside RT_4_, and even the OT genin present only in steamed VG.

In this study, both HPLC-PDA and HPLC-ELSD were developed and validated individually, which could be applied solely in the quantitation of three groups of saponin (PPT, PPD, and OT). Therefore, the QC laboratory could apply one of the two methods for the quality control of VG based on the availability of the PDA/UV detector or ELSD. However, in terms of research (e.g., elucidating the accumulation pattern of saponin throughout ages, the effect of soil and climate on the saponin production), it is mandatory to acquire a comprehensive saponin profile of VG root. Yet, the low sensitivity of the ELSD prevents the quantified measurement of low-level compounds such as G-Rd, as well as other minor compounds in VG, such as vina-ginsenoside R11 or notoginsenoside R4. Similarly, the UV/PDA could not detect V-R2 in the VG extract due to the lower level compared to M-R2. To overcome this obstacle, a dual-detector method integrating a PDA and ELSD could enhance the ability to quantify both chromophore-containing and non-chromophore saponins, which makes the VG chemical profile unique. There have been many studies that have employed the dual-detector approach to detect both chromophoric and non-chromophoric compounds. Ma et al. (2019) used HPLC-PDA-ELSD to identify 29 compounds in the fingerprint profile of Ginkgo biloba leaf extracts, including the terpene trilactones and flavonoid glycosides [18]. A complementary of UV and an ELSD was also used for the detection of 15 compounds in *Stevia rebaudiana bertoni*. Among them, diterpene glycosides were detected by UV at 210 nm, while the commercial additive erythritol was only detected with the ELSD [19]. The integration of an ELSD and UV or PDA detectors could facilitate the comprehensive reveal of the chemical profile of the extracts or natural-product-derived preparations as well as the detection of the additives.

## 5. Conclusions

In this study, QAMS methods using HPLC coupled with a PDA detector or ELSD were developed to quantify five major saponins in VG using G-Rb_1_ and M-R2 as reference standards. The methods exhibited high consistency with the ESM, with SMD below 5%. These cost-effective methods were validated for specificity, linearity, precision, and accuracy, which could be applied widely in the quality control of VG as well as VG-containing products.

## Figures and Tables

**Table 1:** Calculating equations for F_x_ and C_x_.

The Regression Equation and F_x_		Calculate C_x_ (mg/mL)	
HPLC-PDA			
A_x_ = a_x_ × C_x_ + b_x_	(1)	C_x_ = $\frac{A_{x}-b_{x}}{a_{x}}$	(4)
$F_{x}=\frac{A_{x}/C_{x}}{A_{s}/C_{s}}$	(2)	C_x_ = $\frac{A_{x}\times C_{s}}{F_{x}\times A_{s}}$	(5)
$F_{x}=\frac{a_{x}}{a_{s}}$	(3)	C_x_ = $\frac{{(A}_{x}-b_{x}{)\times C}_{s}}{{(A}_{s}-b_{s}{)\times F}_{x}}$	(6)
HPLC-ELSD			
log (A_x_) = a_x_ × log (C_x_) + b_x_	(7)	C_x_ = $10^{(\frac{{\log(A}_{x})-b_{x}}{a_{x}}-3)}$	(10)
$F_{x}=\frac{{\log(A}_{x}{)/\log(C}_{x})}{{\log(A}_{s}{)/\log(C}_{s})}$	(8)	C_x_ = $10^{(\frac{{\log(A}_{x}{)\times \log(C}_{S})}{F_{x}\times {\log(A}_{S})}-3)}$	(11)
$F_{x}=\frac{a_{x}}{a_{s}}$	(9)	C_x_ = $10^{(\frac{{(\log(A}_{x})-b_{x}{)\times \log(C}_{S})}{{(\log(A}_{S})-b_{S}{)\times F}_{x}}-3)}$	(12)

Note: A_x_, A_S_: the peak area of the analyte and single standard (mAU × s) for PDA, (mV × s) for ELSD; C_x_: the concentration of the analyte (mg/mL); C_S_: the concentration of the single standard (mg/mL) for PDA, (μg/mL) for ELSD; a_x_, b_x_: the slope and intercept of the regression Eq.; F_x_: the relative conversion factors.

**Table 2:** The average relative conversion factors for the saponins.

Compound		Average Relative Conversion Factor (F_x_)			
		HPLC-PDA		HPLC-ELSD	
		F_x_ (2)	F_x_ (3)	F_x_ (8)	F_x_ (9)
Single marker G-Rb_1_	G-Rb_1_	1.00	1.00	1.00	1.00
	G-Rg_1_	1.42	1.32	0.94	0.95
	G-Rd	1.08	1.08	N.D	N.D
	M-R2	0.04	0.04	0.93	0.92
	V-R2	N.D	N.D	0.97	0.97
Single marker M-R2	M-R2	1.00	1.00	1.00	1.00
	V-R2	N.D	N.D	1.04	1.05

N.D: not detected.

**Table 3:** Linearity, linear range, LOD, and LOQ of HPLC-PDA and HPLC-ELSD methods.

Compound	Linearity (n = 3)	*R*	Linear Range (mg/mL)	LOD (mg/mL)	LOQ (mg/mL)
HPLC-PDA					
G-Rg_1_	y = 13,426 x + 846,186	0.9998	0.33–1.39	0.055	0.167
M-R2	y = 374 x + 14,146	0.9999	0.55–2.30	0.065	0.197
V-R2	N.D	N.D	N.D	N.D	N.D
G-Rb_1_	y = 10,175 x + 50,838	0.9999	0.09–0.36	0.010	0.030
G-Rd	y = 11,035 x + 17,292	1.0000	0.04–0.17	0.007	0.022
HPLC-ELSD					
G-Rg_1_	y = 1.3722 x + 1.0728	0.9999	0.33–1.39	0.002	0.010
M-R2	y = 1.3223 x + 1.2472	0.9996	0.55–2.30	0.003	0.018
V-R2	y = 1.3942 x + 1.0544	0.9999	0.19–0.81	0.002	0.007
G-Rb_1_	y = 1.4371 x + 0.9450	0.9998	0.09–0.36	0.001	0.002
G-Rd	y = 1.1121 x + 1.3828	0.9937	0.04–0.17	0.014	2.804

N.D: not detected.

**Table 4:** Results of the investigation of intra-day and inter-laboratory precision.

Compound			%RSD (n = 6) Shimadzu and Agilent HPLC-PDA Systems					
			ESM (Equation (4))		QAMS (Equation (5))		QAMS (Equation (6))	
			Shimadzu HPLC System	Agilent HPLC System	Shimadzu HPLC System	Agilent HPLC System	Shimadzu HPLC System	Agilent HPLC System
Single marker G-Rb_1_	G-Rb_1_	Intra-day	1.10	1.89	1.10	1.89	1.10	1.89
		Inter-laboratory	1.89		1.89		1.89	
	G-Rg_1_	Intra-day	0.88	1.97	0.80	1.81	0.88	1.97
		Inter-laboratory	2.86		3.95		2.86	
	G-Rd	Intra-day	1.62	1.68	1.61	1.64	1.62	1.68
		Inter-laboratory	6.48		6.81		6.47	
	M-R2	Intra-day	0.96	0.26	0.92	0.20	0.96	0.26
		Inter-laboratory	1.89		0.65		1.94	
	V-R2	Intra-day	N.D		N.D		N.D	
		Inter-laboratory	N.D		N.D		N.D	
Single marker M-R2	M-R2	Intra-day	0.96	0.26	0.96	0.26	0.96	0.26
		Inter-laboratory	1.89		1.89		1.89	
	V-R2	Intra-day	N.D		N.D		N.D	
		Inter-laboratory	N.D		N.D		N.D	

N.D: not detected.

**Table 5:** Results of the investigation of intra- and inter-day precision.

Compound			Intra-Day and Inter-Day Precision (%RSD) (n = 6), Shimadzu HPLC-ELSD System					
			ESM (Equation (10))		QAMS (Equation (11))		QAMS (Equation (12))	
			Day 1	Day 2	Day 1	Day 2	Day 1	Day 2
Single marker G-Rb_1_	G-Rb_1_	Intra-day	2.70	1.89	2.70	1.89	2.70	1.89
		Inter-day	2.83		2.83		2.83	
	G-Rg_1_	Intra-day	1.76	2.87	1.87	2.64	1.76	2.87
		Inter-day	2.49		2.53		2.49	
	G-Rd	Intra-day	N.D		N.D		N.D	
		Inter-day	N.D		N.D		N.D	
	M-R2	Intra-day	2.44	3.05	2.48	2.80	2.44	3.05
		Inter-day	2.67		2.65		2.67	
	V-R2	Intra-day	2.72	1.44	2.40	1.23	2.72	1.44
		Inter-day	2.12		1.97		2.12	
Single marker M-R2	M-R2	Intra-day	2.44	3.05	2.44	3.05	2.44	3.05
		Inter-day	2.67		2.67		2.67	
	V-R2	Intra-day	2.72	1.44	2.50	1.19	2.72	1.56
		Inter-day	2.12		1.90		2.15	

N.D: not detected.

**Table 6:** The recovery of HPLC-PDA/ELSD methods for the determination of five saponins.

Compound		Recovery (%) (n = 9)					
		HPLC-PDA			HPLC-ELSD		
		ESM (Equation (4))	QAMS (Equation (5))	QAMS (Equation (6))	ESM (Equation (10))	QAMS (Equation (11))	QAMS (Equation (12))
Single marker G-Rb_1_	G-Rb_1_	80.04–95.30	80.04–95.30	80.04–95.30	90.44–101.56	90.44–101.56	90.44–101.56
	G-Rg_1_	85.98–108.45	86.40–108.92	85.98–108.45	92.95–111.62	113.75–134.21	92.95–111.62
	G-Rd	79.37–97.46	73.95–90.78	79.37–97.46	N.D	N.D	N.D
	M-R2	81.75–99.90	81.35–99.45	81.75–99.90	87.69–110.59	160.90–191.90	87.69–110.59
	V-R2	N.D	N.D	N.D	89.07–111.30	103.05–125.74	89.07–111.30
Single marker M-R2	M-R2	81.75–99.90	81.75–99.90	81.75–99.90	87.69–110.59	87.69–110.59	87.69–110.59
	V-R2	N.D	N.D	N.D	89.07–111.30	74.13–92.84	89.07–111.30

N.D: not detected.

**Table 7:** Saponin content and %SMD, determined by HPLC-PDA (F_x_ intra-day, n = 3).

Method	The Mean Saponin Content Was Calculated from a Dried Herb (%)with F_x_ Values According to G-Rb_1_			
	G-Rb_1_	G-Rd	G-Rg_1_	M-R2
ESM (Equation (4))	1.46 ± 0.11	0.96 ± 0.03	3.96 ± 0.26	5.51 ± 0.43
QAMS (Equation (5))	1.46 ± 0.11	0.96 ± 0.03	3.95 ± 0.24	5.56 ± 0.43
QAMS (Equation (6))	1.46 ± 0.11	0.96 ± 0.03	3.96 ± 0.26	5.51 ± 0.43
%SMD (Equations (4) and (5))	0.00 ± 0.00	0.77 ± 0.18	0.34 ± 0.75	0.79 ± 0.28
%SMD (Equation (4) and (6))	0.00 ± 0.00	0.00 ± 0.00	0.00 ± 0.00	0.00 ± 0.00

**Table 8:** Saponin content and %SMD, determined by HPLC-ELSD (F_x_ intra-day, n = 3).

Method	The Mean Saponin Content Was Calculated from a Dried Herb (%)					
	with F_x_ Values According to G-Rb_1_				with F_x_ Values According to M-R2	
	G-Rb_1_	G-Rg_1_	M-R2	V-R2	M-R2	V-R2
ESM (Equation (10))	1.62 ± 0.11	3.99 ± 0.27	5.71 ± 0.38	2.67 ± 0.16	5.71 ± 0.38	2.67 ± 0.16
QAMS (Equation (11))	1.62 ± 0.11	4.55 ± 0.33	6.79 ± 0.47	2.93 ± 0.19	5.71 ± 0.38	2.52 ± 0.15
QAMS (Equation (12))	1.62 ± 0.11	3.99 ± 0.27	4.13 ± 0.26	2.68 ± 0.16	5.71 ± 0.38	2.67 ± 0.16
%SMD (Equations (10) and (11))	0.00 ± 0.00	13.98 ± 1.05	18.92 ± 0.90	9.80 ± 1.04	0.00 ± 0.00	5.77 ± 0.69
%SMD (Equations (10) and (12))	0.00 ± 0.00	0.00 ± 0.00	27.65 ± 0.48	0.51 ± 0.01	0.00 ± 0.00	0.00 ± 0.00

**Table 9:** Saponin content and %SMD determined by HPLC-PDA (F_x_ inter-day, n = 3).

Method	The Mean Saponin Content Was Calculated from a Dried Herb (%)with F_x_ Values According to G-Rb_1_			
	G-Rb_1_	G-Rd	G-Rg_1_	M-R2
ESM (Equation (4))	1.41 ± 0.24	0.94 ± 0.07	3.78 ± 0.22	5.69 ± 0.40
QAMS (Equation (5))	1.41 ± 0.24	0.93 ± 0.07	3.76 ± 0.21	5.71 ± 0.39
QAMS (Equation (6))	1.41 ± 0.24	0.94 ± 0.07	3.78 ± 0.22	5.70 ± 0.40
%SMD (Equations (4) and (5))	0.00 ± 0.00	0.63 ± 0.25	0.42 ± 0.88	0.40 ± 0.30
%SMD (Equations (4) and (6))	0.00 ± 0.00	0.01 ± 0.00	0.01 ± 0.00	0.11 ± 0.00

**Table 10:** Saponin content and SMD, determined by HPLC-ELSD (F_x_ inter-day, n = 3).

Method	The Mean Saponin Content Was Calculated from a Dried Herb (%)					
	with F_x_ Values According to G-Rb_1_				with F_x_ Values According to M-R2	
	G-Rb_1_	G-Rg_1_	M-R2	V-R2	M-R2	V-R2
ESM (Equation (10))	1.58 ± 0.10	3.91 ± 0.24	6.00 ± 0.34	2.75 ± 0.15	6.00 ± 0.34	2.75 ± 0.15
QAMS (Equation (1))	1.58 ± 0.10	4.46 ± 0.29	7.02 ± 0.34	2.97 ± 0.19	6.00 ± 0.34	3.31 ± 0.15
QAMS (Equation (2))	1.58 ± 0.10	3.91 ± 0.24	6.00 ± 0.34	2.75 ± 0.15	6.00 ± 0.34	2.75 ± 0.15
%SMD (Equations (10) and (1))	0.00 ± 0.00	14.20 ± 0.25	17.01 ± 1.07	8.32 ± 0.42	0.00 ± 0.00	20.55 ± 0.45
%SMD (Equations (10) and (2))	0.00 ± 0.00	0.00 ± 0.00	0.01 ± 0.00	0.03 ± 0.00	0.00 ± 0.00	0.28 ± 0.71

**Table 11:** SMD of two QAMS (F_x_-Slope) methods, intra- and inter-day.

**HPLC-PDA**	**F_x_**	**The mean saponin content was calculated on a dried herb (%)**			
		**with F_x_-Slope values according to G-Rb_1_**			
		**G-Rb_1_**	**G-Rd**	**G-Rg_1_**	**M-R2**
	**Intra-day (n = 3)**	1.46 ± 0.24	0.96 ± 0,07	3.96 ± 0,56	5.51 ± 0,95
	**Inter-day (n = 3)**	1.41 ± 0.11	0.94 ± 0,07	3.78 ± 0,25	5.69 ± 0,45
	**%SMD**	3.71 ± 0.48	1.87 ± 1.07	4.66 ± 0.42	3.31 ± 0.14
**HPLC-ELSD**	**F_x_**	**With F_x_-Slope values according to G-Rb_1_**		**With F_x_-Slope values according to M-R2**	
		**G-Rb_1_**	**G-Rg_1_**	**M-R2**	**V-R2**
	**Intra-day (n = 3)**	1.62 ± 0.25	3.99 ± 0.60	5.71 ± 0.83	2.67 ± 0.35
	**Inter-day (n = 3)**	1.58 ± 0.25	3.91 ± 0.59	6.00 ± 0.86	2.75 ± 0.36
	**%SMD**	2.44 ± 0.17	2.01 ± 0.24	5.00 ± 0.37	3.13 ± 0.90

## Data Availability

The original contributions presented in this study are included in the article. Further inquiries can be directed to the corresponding author.

## Supplementary Materials

The following supporting information can be downloaded at: https://www.mdpi.com/article/10.3390/metabo15070419/s1, Table S1: Retention time of the quantified compounds.

## Abbreviations

The following abbreviations are used in this manuscript:

PDA	Photodiode Array;
ELSD	Evaporative Light Scattering Detector;
QAMS	Quantitative Analysis of Multi-components by Single-marker;
VG	Vietnamese Ginseng;
OT	Ocotillol;
EMS	External Standards Method;
Eq.	Equation.
