# Supplementary material for: Simultaneous Quantification of Main Saponins in Panax vietnamensis by HPLC-PDA/ELSD Using the Quantitative Analysis of Multi-Components by Single-Marker Method
Source: Metabolites. 2025 Jun 20;15(7):419. doi: 10.3390/metabo15070419 (PMC12298259; doi:10.3390/metabo15070419)
Supplement: Supplementary file 1 [file metabolites-15-00419-s001.zip › metabolites-3627611-supplementary.pdf]

Table S1. Retention time of the quantified compounds

Compounds	HPLC-PDA	HPLC-ELSD
G-Rg1	11.49	12.10
M-R2	13.74	14.43
V-R2	35.97	36.34
G-Rb1	41.69	42.04
G-Rd	55.86	56.37
